# The roles of FGF21 and GDF15 in mediating the mitochondrial integrated stress response

**DOI:** 10.3389/fendo.2023.1264530

**Published:** 2023-09-25

**Authors:** Jayashree Jena, Luis Miguel García-Peña, Renata O. Pereira

**Affiliations:** Fraternal Order of Eagles Diabetes Research Center and Division of Endocrinology and Metabolism, Roy J. and Lucille A. Carver College of Medicine, University of Iowa, Iowa City, IA, United States

**Keywords:** mitochondrial stress, FGF21, GDF15, integrated stress response, metabolic health, energy balance

## Abstract

Various models of mitochondrial stress result in induction of the stress-responsive cytokines fibroblast growth factor 21 (FGF21) and growth differentiation factor 15 (GDF15). This is an adaptive mechanism downstream of the mitochondrial integrated stress response frequently associated with improvements in systemic metabolic health. Both FGF21 and GDF15 have been shown to modulate energy balance and glucose homeostasis, and their pharmacological administration leads to promising beneficial effects against obesity and associated metabolic diseases in pre-clinical models. Furthermore, endogenous upregulation of FGF21 and GDF15 is associated with resistance to diet-induced obesity (DIO), improved glucose homeostasis and increased insulin sensitivity. In this review, we highlight several studies on transgenic mouse models of mitochondrial stress and will compare the specific roles played by FGF21 and GDF15 on the systemic metabolic adaptations reported in these models.

## Introduction

Chronic mitochondrial stress is associated with the activation of stress response pathways such as the mitochondrial unfolded protein response (UPR^mt^) (1) and the integrated stress response (ISR) (2) in various tissues such as liver, adipose tissue and skeletal muscle. This is believed to be an adaptive mechanism to mitigate stress at the cellular level, but is also associated with cell non-autonomous mechanisms via the secretion of mitokines (3).

Metabolic stress and changes in mitochondrial membrane potential can result in mitochondrial dysfunction, thereby inducing the mitochondrial stress response (1). The UPR^mt^ mediates retrograde mitochondria to nucleus signaling to alleviate mitochondrial stress (4). In mammals, it has been shown that the UPR^mt^ is strongly coupled with the ISR (5). The ISR can be induced by four different eukaryotic initiation factor 2 alpha (eIF2α) kinases that act as early responders to disturbances in cellular homeostasis (2). Once activated, the ISR leads to phosphorylation of eIF2α and induction of its main effector, the activating transcription factor 4 (ATF4), which coordinates activation of transcriptional programs aiming at restoring cellular homeostasis. The ISR can promote UPR^mt^ activation, thereby reducing the stress on mitochondrial chaperones and proteases, and attenuating mitochondrial dysfunction (5). ATF4 can also mediate the transcription of fibroblast growth factor 21 (FGF21) and growth differentiation factor 15 (GDF15) (6–9), mitokines that can act distally to regulate systemic metabolic homeostasis (10, 11).

FGF21 and GDF15 can also be induced under physiological stress conditions, such as in response to an obesogenic diet (12, 13), exercise (14, 15) and cold exposure (16, 17). Furthermore, pharmacological administration of FGF21 or GDF15 ameliorates obesity and related metabolic complications by improving energy and glucose homeostasis (18, 19). Nonetheless, there are several yet unresolved questions regarding the endogenous roles played by these cytokines in response to physiological versus mitochondrial stress.

In this review, we will focus on the systemic metabolic adaptations mediated by endogenous FGF21 and GDF15 induction in response to mitochondrial stress and activation of the mitochondrial stress response. We will summarize various transgenic mouse models harboring mitochondrial defects of different etiologies and in different tissues but resulting in similar improvements in metabolic health. Finally, we will discuss downstream mechanisms that are predominantly regulated by either FGF21 or GDF15 and their possible synergistic effects on systemic metabolism.

## FGF21 and its roles in metabolism

FGF21 is a member of the fibroblast growth factor (FGF) family that can be released into the circulation to act as a hormone. High FGF21 levels have been reported in various metabolic diseases, including obesity, fatty liver disease, and diabetes (20, 21). In both, rodents (22) and humans (23), circulating levels of FGF21 are derived primarily from the liver under physiological conditions, however *Fgf21* mRNA can be detected in numerous tissues including the pancreas, muscle, and adipose tissues (24, 25). Circulating levels of FGF21 are low under baseline conditions and are markedly induced by numerous cellular and nutritional stress signals (21). Depending on the metabolic state, induction of FGF21 instructs the system to reestablish homeostasis through actions on multiple tissues (21). In the context of metabolism, FGF21 has been proposed to function in an endocrine manner as a ‘master sensitizer’ of specific hormonal signals that maintain energy balance via the regulation of glucose and lipid metabolism. Furthermore, previous studies have established a role for FGF21 in enhancing insulin sensitivity, increasing energy homeostasis, decreasing hepatic triglycerides, and regulating macronutrient preferences (21, 26). The multiple metabolic effects of FGF21 are mediated by both its central and peripheral actions, and by its fine-tuning of inter-organ metabolic crosstalk (26).

Besides its endogenous roles, pharmacological administration of FGF21 can decrease plasma glucose levels by more than 50% in animal models with genetically induced and diet-induced obesity (DIO) mainly through peripheral glucose disposal (21). Furthermore, prolonged administration of FGF21 or FGF21 analogs has important metabolic effects, including a marked decrease in body weight in rodents and non-human primates, and more modest effects on body weight in humans (10, 27, 28). In the context of obesity, pharmacological administration of FGF21 to obese rodents reverses diabetes and obesity through increasing energy expenditure (28, 29). Moreover, prolonged FGF21 administration to DIO mice significantly decreases body weight, adiposity, and hepatic triglycerides and cholesterol. FGF21 also reverses plasma hyperglycemia and hypertriglyceridemia and increases insulin sensitivity (28). In individuals with obesity and type 2 diabetes mellitus, FGF21 analogs alleviate dyslipidemia and increase adiponectin levels, but have minimal effects on glycemic control, thereby highlighting interspecies differences in the actions of FGF21 (26). Similarly, in patients with non-alcoholic steatohepatitis, FGF21 analogues ameliorate hepatic steatosis, liver stiffness and biomarkers of liver fibrosis (26), however the long-term effects of FGF21 in clinical outcomes remain unknown.

## GDF15 and its role in metabolism

Growth differentiation factor 15 (GDF15), also known as macrophage inhibitory cytokine-1 [MIC-1] (12), is a stress-induced cytokine originally classified as a divergent member of the transforming growth factor beta (TGF-β) superfamily (30). GDF15 is synthesized as pro-GDF15 dimer in the cytoplasm and is subsequently cleaved and secreted as mature GDF15 (25kDa) into the blood stream (31). GDF15 is expressed in several tissues including liver (12, 32), lungs (33), kidney (34), and adipose tissue (12, 17). Levels of mature circulating GDF15 can be measured and are normally very low in humans under normal physiological conditions (35, 36). However, GDF15 levels are markedly elevated in various pathological conditions including cancer (37), inflammatory diseases (38), cardiovascular disease (39, 40), obesity (12) and mitochondrial dysfunction (41).

Glial cell–derived neurotrophic factor (GDNF) family receptor α-like protein (GFRAL) has been recently identified as GDF15’s receptor (18, 42). GFRAL’s expression is restricted to neurons of the area postrema and solitary tract nucleus in the brain (43). Mature GDF15 binds to its receptor GFRAL in the brain inducing phosphorylation of RET (tyrosine kinase co-receptor) and other intracellular signaling molecules, such as AKT and ERK1/2, thereby controlling appetite (12). Furthermore, studies also suggest mature GDF15 may affect several metabolic pathways independently of the GFRAL/RET receptor pathway and can modulate metabolism independently of changes in food intake. Indeed, systemic overexpression of GDF15 prevents obesity and insulin resistance by modulating metabolic activity and enhancing the expression of thermogenic and lipolytic genes in brown adipose tissue (BAT) and white adipose tissue (WAT) (44–46). Some of these effects were independent of changes in food intake (43).

Obesity is also associated with increased serum concentrations of GDF15. An increase of up to 10-fold in serum GDF15 levels was observed in obese mice (12). Several studies have reported the role of GDF15 towards reducing food intake, body weight, and adiposity and improving glucose tolerance under normal and obesogenic diets (12, 45, 47). Indeed, GDF15 and GFRAL knockout mice fed a high-fat diet (HFD) display a slight increase in fat depots and body weight when compared to their wild-type counterparts (43, 48). Conversely, GDF15 overexpression is associated with leanness and other improved metabolic parameters in mice (44–46).

Besides its endogenous roles, pharmacological administration of GDF15 has gained significant interest owing to its role as an appetite suppressor and a regulator of energy homeostasis (18, 49). Pre-clinical studies administering recombinant GDF15 in rodents and non-human primates revealed the potential of GDF15 for the treatment of metabolic disorders such as obesity and diabetes. GDF15 treatment potently reduces food intake in ob/ob mice (50), and in non-human primates with spontaneous obesity (18). Several studies have shown that treatment with recombinant-GDF15 attenuates obesity and improves glycemic control through GFRAL-dependent suppression of food intake (18, 42). Furthermore, administration of a long-acting GDF15 molecule to non-human primates maintained the decrease in appetite and body weight for 4 weeks, suggesting this molecule might be used for chronic treatment of obesity (51). In addition to changes in food intake and body weight, recombinant GDF15 also reduces liver steatosis and improves glycemic control via GFRAL in genetically induced and DIO mouse models (45, 46). Noteworthy, besides reducing food intake, treatment with recombinant GDF15 reduces adiposity and corrects metabolic dysfunction in obese mice by increasing thermogenesis in adipocytes (35), and by increasing energy expenditure via increased adrenergic stimulation and futile calcium cycling in skeletal muscle through GFRAL signaling (52). Recent studies also uncovered roles for GDF15 in mediating the beneficial effects of compounds with anti-obesity properties, including metformin (53), capsaicin (54), resveratrol (55) and conjugated linoleic acid (56). Furthermore, GDF15 may also interact with other appetite regulators such as leptin to regulate body weight and adiposity (57). Lastly, a GLP-1/GDF15 dual agonist effectively lowers body weight and caloric intake in various animal models, including obese non-human primates (58).

These promising pre-clinical studies have now been extended to clinical trials. Indeed, results from a recently completed clinical trial using a new long-acting GDF15 receptor agonist show significant reduction in body weight in rodents and in non-human primates. However, despite displaying an acceptable safety profile and reducing food intake in humans, body weight was only modestly affected (49). These interesting results highlight potential differences in GDF15 signaling and mechanisms of action between rodents, non-human primates and human subjects. Furthermore, this suggests that additional GDF15-mediated mechanisms that might be independent of GFRAL signaling, or food intake reduction are likely required to promote weight loss in humans.

## FGF21 and GDF15 as downstream modulators of the mitochondrial integrated stress response

**Figure 1:**
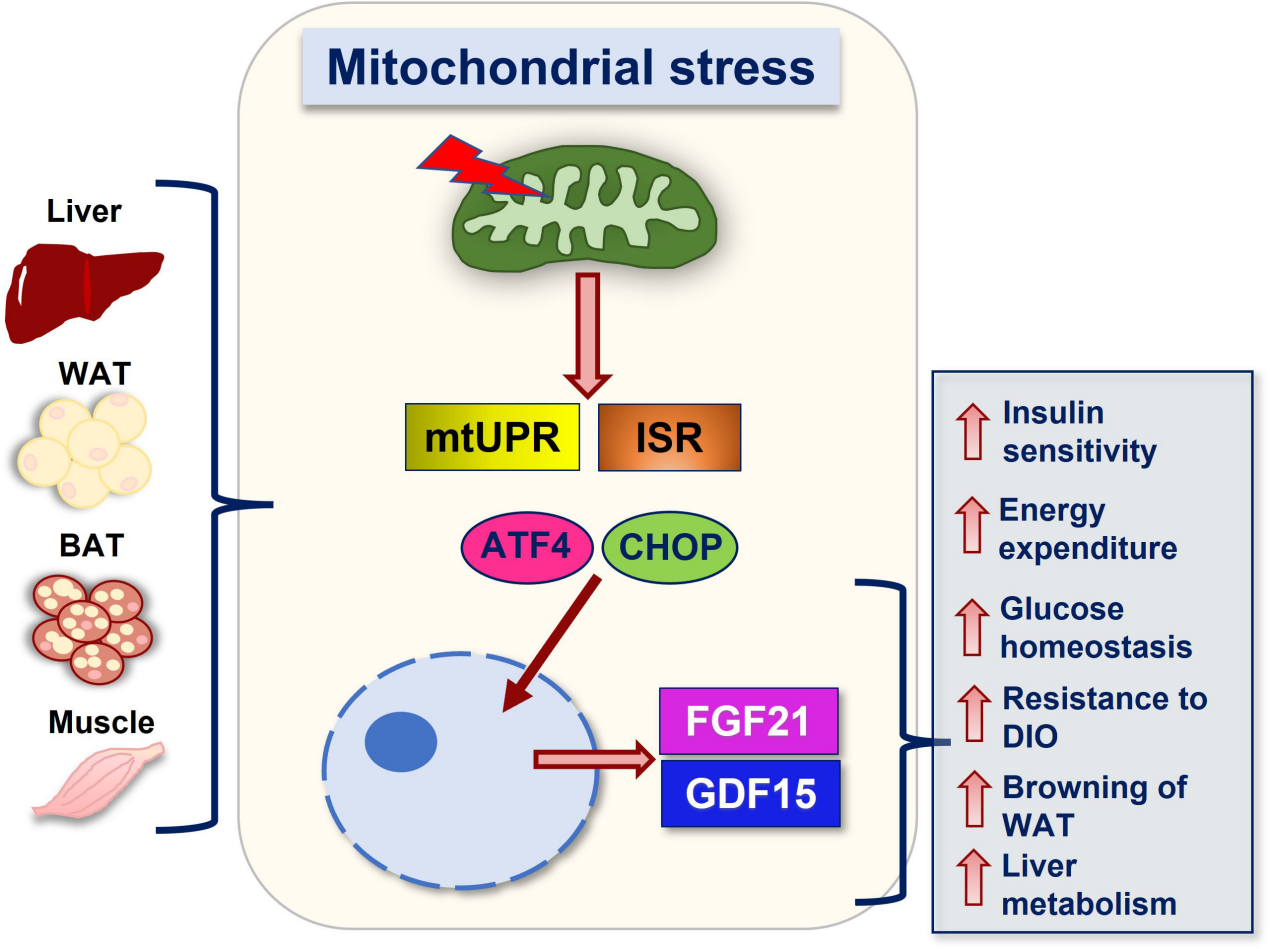
Summary of FGF21 and GDF15 mediated actions downstream of mitochondrial stress. Mitochondrial stress of various etiologies and in various tissues lead to the activation of stress response pathways such as the mtUPR and the ISR, resulting in the translation of transcription factors such as ATF4 and its downstream target CHOP, which promote induction of FGF21 and GDF15, respectively. Secretion of these factors into the circulation mediates metabolic adaptations in response to mitochondrial stress. Together, these adaptations promote improvements in systemic metabolic health, including increases in energy expenditure, resistance to diet-induced obesity (DIO), improved insulin sensitivity and glucose homeostasis, attenuated hepatic steatosis, and increased browning. Abbreviations: WAT (white adipose tissue), BAT (brown adipose tissue), UPR^mt^ (mitochondrial unfolded protein response), ISR (integrated stress response), ATF4 (activating transcription factor 4), CHOP (C/EBP homologous protein), FGF21 (fibroblast growth factor 21) and GDF15 (growth differentiation factor 15).

FGF21 and GDF15 can be induced in response to mitochondrial stress, and as such are recognized as mitokines (59, 60). Mitokines are signaling molecules that enable communication between local mitochondrial stress and distant cells and tissues, that can be strongly induced in mouse models of metabolic dysregulation and mitochondrial dysfunction (59). Mitochondrial dysfunction of various etiologies, such as defective mitochondrial oxidative phosphorylation (OxPhos) and coupling efficiency (59, 61), impaired quality control (46) or dynamics (6, 62, 63) activate the UPR^mt^ and the ISR, leading to increased phosphorylation of eIF2α (2). eIF2α phosphorylation inhibits overall translation, while selectively allowing for the translation of stress responsive proteins such as the activating transcription factor 4 (ATF4). Downstream of eIF2α, ATF4 directly induces *Fgf21* transcription and via induction of C/EBP homologous protein (CHOP) it indirectly induces *Gdf15* expression (59, 64) (**Figure 1**).

Several mouse models of mitochondrial stress have shown an upregulation of FGF21 as a protective mechanism against mitochondrial injury and metabolic dysregulation. Ectopic expression of the uncoupling protein 1 (UCP1) in skeletal muscle *in vivo* (UCP1-Tg) leads to induction of FGF21, which is required to increase browning of white adipose tissue (WAT), with only mild effects on the metabolic phenotype of these mice (9, 59). In mice lacking UCP1 in brown adipose tissue (BAT), the main effector in canonical BAT thermogenesis, induction of FGF21 promotes resistance to DIO (61). In models of disrupted mitochondrial dynamics in skeletal muscle (62), BAT (6) and liver (63), secretion of FGF21 resulted in protection against DIO and improved metabolic phenotypes. Genetic impairment of adipocytes OxPhos function *in vivo* also protects mice from DIO and insulin resistance, with ablation of FGF21 in this model leading to increased body weight and adiposity, and hepatic steatosis after 8 weeks on high-fat diet (HFD) (41). ATG7 deletion selectively in skeletal muscle inhibits mitophagy and induces FGF21 as a mitokine, leading to protection from obesity and insulin resistance (65). Another model of impaired mitophagy also showed upregulated *Fgf21* expression, and a role for ISR-mediated secretion of FGF21 in promoting thermogenic remodeling of adipose tissue (66). Furthermore, several studies of gain- and loss-of-function indicate that FGF21 is a key metabolic mediator to improve compromised mitochondrial function and reduce inflammation and apoptosis in skeletal muscle (67).

Various transgenic mouse models of mitochondrial dysfunction also result in GDF15 induction in a tissue-specific manner, leading to improved systemic metabolic homeostasis (60, 68). Elevated GDF15 expression in and secretion from skeletal muscle has been described in human subjects with mitochondrial myopathies (69–71). Furthermore, mouse models of selective mitochondrial dysfunction in muscle have higher levels of circulating GDF15, which is associated with improved aspects of systemic metabolism. Indeed, mice with muscle-specific deletion of a component of mtDNA ribosomes, *Crif-1*, have impaired oxidative phosphorylation and upregulation of *Gdf15* expression in and secretion from muscle via activation of a UPR^mt^-ATF4-CHOP axis. This increase in GDF15 levels was required to promote resistance to DIO and insulin resistance, due to an increase in lipolysis in adipocytes and hepatocytes (60). In another model of mitochondrial dysfunction caused by *Ant1* deficiency in muscle, *Gdf15* levels were highly up regulated (72). Moreover, UCP1-tg mice also leads to increased GDF15 circulating levels. Deletion of either *Fgf21* or *Gdf15* in the UCP1-tg background showed that FGF21 was of minor importance for the metabolic adaptations of this particular mouse model (9), whereas ablation of *Gdf15* led to a progressive body mass increase due to an accumulation of body fat, and abolished the increased insulin sensitivity reported in this mouse model. These effects were also associated with day-time restricted anorexia, which was dependent on GDF15 (7).

Mice with liver-specific mitochondrial stress induced by a loss of function of *Crif-1* have aberrant OxPhos and activated UPR^mt^, increasing the secretion of GDF15 and FGF21 as mitokines. By using whole body GDF15 or FGF21 knock out mouse models in conjunction with *Crif-1* deletion in liver, the authors concluded that GDF15 is necessary to regulate body mass and fat mass and to prevent diet-induced hepatic steatosis, whereas FGF21 is requited to improve insulin sensitivity, energy expenditure and thermogenesis in WAT (32).

Furthermore, adipocyte-specific deletion of *Crif-1* in mice decreases adipocyte OxPhos function and induces FGF21 and GDF15 as mitokines, thereby promoting resistance to DIO, and improving glucose homeostasis. Long-term induction of GDF15 in this model was required to attenuate the progression of obesity by increasing energy expenditure, while FGF21 did not affect energy expenditure, but remarkably ameliorated DIO and insulin resistance (41). Finally, a report by Miyake et al. shows that induction of GDF15 secretion mediated by activation of the integrated stress response (ISR) in adipocytes reduces food intake via GFRAL signaling and decreases body weight in mice fed HFD (64).

## Mitochondrial stress in the pathogenesis of metabolic diseases

It is important to note that, although chronic mild mitochondrial stress can lead to adaptive responses that result in improved metabolic health, unresolved mitochondrial stress is central to the pathogenesis of many major metabolic disorders such as obesity, insulin resistance and type 2 diabetes (4, 73). During OxPhos, in addition to generating ATP, mitochondria also generate reactive oxygen species (ROS) as toxic by-products. In excess, ROS may damage mitochondrial and cellular DNA, proteins, lipids and other molecules, contributing to mitochondrial dysfunction (74).

Mitochondrial dysfunction has been shown to be associated with insulin resistance in various tissues including skeletal muscle (75), liver (76) and adipose tissue (77). In skeletal muscle, impaired mitochondrial oxidative capacity, reduced ATP production rates and increased ROS levels have all been reported as contributing factors in the pathogenesis of insulin resistance (4). Impaired mitochondrial β-oxidation is found in patients with nonalcoholic fatty liver disease (NAFLD), contributing to hepatic steatosis, liver injury (78, 79) and fibrosis (80). Mitochondria are also crucial for adipose tissue function (73), therefore, unsurprisingly, impaired mitochondrial function and dynamics disrupts adipocyte homeostasis (77, 81) and is a central driver for obesity, inflammation and associated metabolic disorders (82).

## Discussion

Both GDF15 and FGF21 are central endocrine regulators of systemic energy metabolism and glucose homeostasis with robust therapeutical potential for the treatment of obesity and associated metabolic diseases (10, 18). Pre-clinical studies suggest that GDF15 acts predominantly on reducing energy intake (42, 51), whereas FGF21 might play a primary role on increasing energy expenditure (10, 21). Nonetheless, recent clinical trials were unable to show a robust effect on weight loss, even though other metabolic parameters seem to be ameliorated by FGF21 and GDF15 treatment (49, 83, 84). Therefore, further studies will be required to determine the primary targets and fundamental mechanisms of actions of both factors in humans so that more effective therapies can be developed.

In mouse models of mild mitochondrial stress, chronically elevated FGF21 and GDF15 provide resistance to DIO and insulin resistance and ameliorate diet-induced hepatic steatosis and glucose intolerance (6, 32, 62). These effects are likely caused by the lifelong induction of a catabolic state in these models. However, induction of FGF21 levels in adult obese mice, by inducible deletion of the mitochondrial fusion protein optic atrophy 1 (OPA1) in skeletal muscle, was able to reverse DIO, suggesting that induction of this pathway after the onset of obesity may still exert beneficial metabolic effects (62). Noteworthy, GDF15 and FGF21 play divergent roles in regulating energy metabolism and glucose homeostasis as part of the mitochondrial stress response. These different roles are dictated by the type of mitochondrial stress, the tissue directly affected by the stress and the metabolic state of the animal. Together, studies support a predominant role for GDF15 rather than FGF21 in promoting resistance to DIO in response to mitochondrial stress and suggests a role for GDF15 in promoting browning during DIO (32). Conversely, FGF21 is required to increase insulin sensitivity, energy expenditure and UCP1-mediated thermogenesis in inguinal white adipose tissue (iWAT) of regular chow-fed mice (6, 32). However, whether FGF21 and GDF15 interact to modulate energy balance and glucose homeostasis in response to mitochondrial stress remains to be investigated.

Finally, while our understanding of the role of endogenous FGF21 and GDF15 in the regulation of metabolic homeostasis in response to mitochondrial stress has advanced in the recent years with the characterization of various mouse models, several outstanding questions remain to be addressed. Although studies have shown that the pharmacological actions of FGF21 are primarily mediated by its receptor in the brain and in adipose tissues, to date, the only GDF15 receptor identified is strictly expressed in discrete areas of the brain. Nonetheless, studies suggest GDF15 may exert some of its effects independently of GFRAL via activation of yet non-identified receptors. Moreover, the role of these cytokines in response to physiological stressors such as exercise and cold exposure remain to be assessed. Better understanding of the roles of endogenous FGF21 and GDF15 in health and disease might lead to the development of new therapies to treat metabolic disorders.

## Author contributions

JJ: Writing – review & editing, Writing – original draft. LG-P: Writing – original draft, Writing – review & editing. RP: Writing – review & editing, Conceptualization, Funding acquisition, Project administration, Resources, Supervision.
